# Exploring the ElectroRetinoGraphy as a biomarker for predicting and monitoring therapeutic response to antidepressants in major depressive disorder: study protocol for the MESANTIDEP trial

**DOI:** 10.3389/fpsyt.2025.1501166

**Published:** 2025-04-25

**Authors:** Marie de Deus, Charlotte Petit, Marie Moulard, Eve Cosker, Naoual Mellouki Bendimred, Éliane Albuisson, Julia Maruani, Pierre-Alexis Geoffroy, Thomas Schwitzer

**Affiliations:** ^1^ Pôle Hospitalo-Universitaire de Psychiatrie d’Adulte et d’Addictologie du Grand Nancy, Centre Psychothérapique de Nancy, Laxou, France; ^2^ Délégation à la Recherche Clinique et à l'Innovation (DRCI), Unité de Méthodologie, Data Management et Statistique (UMDS), Centre Hospitalier Régional et Universitaire (CHRU) de Nancy, Nancy, France; ^3^ Département de Psychiatrie et Addictologie, Assistance publique – Hôpitaux de Paris (AP-HP), Hôpital Bichat Claude-Bernard, Paris, France; ^4^ Centre ChronoS, GHU Paris Nord, Département Médico-Universitaire Département Médico-Universitaire (DMU) Neurosciences, Secteur G22, Hôpital Bichat Claude-Bernard, Paris, France; ^5^ Université Paris Cité, NeuroDiderot, Inserm, Paris, France

**Keywords:** antidepressant, ElectroRetinoGraphy, major depressive disorder, precision psychiatry, retinal function, SSRI, α2 antagonist

## Abstract

**Background:**

Major depressive disorder (MDD) is a frequent and highly debilitating condition for which current antidepressant treatments show limited effectiveness. In addition, their implementation requires one or more trial-and-error processes, which involves months of untreated illness. Achieving faster efficacy by identifying the most adapted treatment for each patient as the first line treatment could significantly reduce MDD-related morbidity and mortality while enhancing patients’ quality of life. To achieve this goal, there is a need to identify markers for predicting and monitoring therapeutic response to antidepressants.

**Methods:**

The MESANTIDEP study is designed to identify electroretinographic (ERG) biomarkers that can predict the therapeutic response at 12 weeks to the two main classes of antidepressants prescribed as first-line treatments for MDD: Selective Serotonin Reuptake Inhibitors (SSRIs) and alpha-2 adrenergic receptor antagonists (α2-antagonists). Secondly, the study aims to explore the relationship between ERG measurements and therapeutic response at 6 and 12 weeks in MDD patients treated with SSRIs or α2-antagonists. To this end, patients diagnosed with MDD and needing to start an antidepressant from the SSRI or α2-antagonist classes will be enrolled. At the inclusion visit, prior to starting their antidepressant treatment, patients will undergo various assessments, including clinical and sleep questionnaires, as well as ERG tests. Patients will initiate their antidepressant treatment the day after the inclusion visit. Subsequent evaluations, identical to those at baseline, will be conducted 6 and 12 weeks afterwards to monitor therapeutic response to antidepressants.

**Discussion:**

The MESANTIDEP study will contribute to identify ERG markers predicting and monitoring the therapeutic response to antidepressants. If such markers are highlighted, it is intended to help clinicians in the treatment management of MDD patients. ERG measurements being easy to perform and accessible to all, they could be integrated into a multimodal treatment approach in routine clinical practice. It would enable more rapid therapeutic intervention tailored to each patient could reduce the functional impact of the disease, improve patients’ quality of life, and decrease MDD-associated morbidity and mortality.

**Clinical Trial Registration:**

Clinicaltrials.gov, identifier NCT06532604.

## 1 Introduction

### 1.1 Background

Major Depressive Disorder (MDD) is a chronic and frequent disorder affecting approximatively 3.8% of the world’s population (1). It is characterized by at least one major depressive episode without mania or hypomania (2). MDD causes physical (3–5) and functional disabilities (6) leading to a significant negative impact on the quality of life of MDD patients. Consequently, this pathology the second-leading cause of Years Lived with Disability among all chronic diseases (7) and the single largest contributor to non-fatal health loss worldwide (1).

MDD is therefore a major public health problem but difficulties are encountered in their therapeutic care. For moderate to severe depression, antidepressants are recommended as first-line treatment (8). Among them, the most frequently prescribed are Selective Serotonin Reuptake Inhibitors (SSRIs) (9) - targeting exclusively serotonin reuptake - followed by other modern agents (10) such as alpha-2 adrenergic receptor antagonists (α2-antagonists) - targeting α_2_ adrenergic receptor. However, antidepressants are not always effective, with only 57.5% of MDD patients receiving antidepressants treatment rating them as very effective and 30.2% as somewhat effective (10). When they are effective, it often occurs after a certain delay (11, 12) or even after numerous courses of different antidepressants (11, 13). Currently, no relevant routine biomarkers are available to guide treatment management. The discovery and validation of such markers could guide practitioners in their management treatment and thus improve the prescription of antidepressants (14).

As the exploration of brain function remains complex, these biomarkers could be found using indirect techniques such as electrophysiology. Interestingly, the retina is considered as a window to the brain (15) since the retina and the brain share a common neuroectodermal origin (16). Thus, they have similar properties in terms of neurochemistry and neuroanatomy (16). One way to explore this neurochemistry in the retina is ElectroRetinoGraphy (ERG). Promising human studies using this technique have shown that the amplitudes and implicit times of P50 in Pattern ERG (PERG) as well as a- and b-waves in full-field ERG (ffERG) under scotopic and photopic condition may differ - increasing or decreasing - according to the therapeutic class of antidepressants administered (17). Therefore, these studies pave the way for precision psychiatry, where each MDD patient could benefit from an adapted treatment thanks to their ERG profile (18). Indeed, ERG measurements could provide reliable biomarkers for predicting and monitoring antidepressant responses in patients with MDD to guide treatment management.

### 1.2 Study aims and hypotheses

In this context, the main aim of the MESANTIDEP study is to evaluate the differences in ERG measurements between responder and non-responder MDD patients to the two main therapeutic classes of antidepressants prescribed as first-line treatments for MDD - SSRIs and α2-antagonists. Secondarily, we will study the correlation between ERG measurements and follow-up of therapeutic response at 6 and 12 weeks (according to the widely used and validated hetero-assessment Montgomery-Asberg Depression Rating Scale (MADRS) score, sleep quality and anxiety level) in MDD patients treated with SSRIs or α2-antagonists.

Our main hypothesis is that electrophysiological data measured in ERG before the initiation of antidepressant treatment differ between responders and non-responders to the prescribed antidepressant class. These differences can be used to identify biomarkers that predict therapeutic responses to SSRI and α2-antagonists. Our second hypothesis is that there will be a correlation between therapeutic response - MADRS score, sleep quality and anxiety level - and ERG after 6 and 12 weeks of treatment. Thus, ERG measurements can be used to identify biomarkers for monitoring SSRI and α2-antagonist responses. Both biomarkers could be applied in everyday practice to improve MDD treatment management.

## 2 Methods and analysis

### 2.1 Study design

The MESANTIDEP study takes place at the Nancy Psychotherapeutic Center and at the Bichat Claude-Bernard hospital in France. It is a longitudinal prospective cohort, open-label and non-randomized comparative multicenter study applied in psychiatry and neuroscience. This research includes two groups of adult MDD patients: a group of patients with a prescription of a SSRI and a group of patients with a prescription of an α2-antagonist. No blinding procedure is planned in this research.

The MESANTIDEP study protocol was reviewed and approved by the French ethics committee (Comité de Protection des Personnes, Sud-Méditerranée V) under the reference 2024/46. The study is also registered at Clinicaltrials.gov under the number NCT06532604. All participants in this research will provide their written informed consent before the start of any clinical interview, assessment or measurement.

### 2.2 Setting and participant recruitment

This study will include two groups of participants. A group of MDD patients with a prescription of a SSRI and a group of MDD patients with a prescription of an α2-antagonist. The antidepressants included are the ones authorized and prescribed in France, i.e. Citalopram, Escitalopram, Fluvoxamine, Paroxetine, Sertraline and Fluoxetine for the SSRI group; Mirtazapine and Mianserine for the α2-antagonist group.

Patients eligible for the study will be recruited from the outpatient population followed at the Nancy Psychotherapeutic Center (France), at the Bichat Claude-Bernard hospital (France) or from outpatients of physicians in private practice near the recruitment center. Doctors will be informed of the study and may refer their patients. Posters and flyers will be distributed within the Nancy Psychotherapeutic Center and in private practices to inform patients about the possibility of participating in the study. External communication - through newspapers, radio and social media - will also be considered if patient recruitment is difficult.

A first telephone contact with interested participants will allow to present the study to the patients, verify the main eligibility criteria and set an appointment for the inclusion visit. Given the lack of urgency in introducing an antidepressant due to its delayed onset of action, eligible participants with a MDD will be met within 48 hours of prescription. During this appointment, the patient is provided with more detailed oral and written information about the main study. In the event that the patient wishes to participate, they give their written informed consent. The patient is subsequently screened for inclusion and exclusion criteria. If the patient meets all the criteria, they are included in the study and can start their treatment after inclusion.

### 2.3 Inclusion and exclusion criteria

The inclusion criteria for patients are as follows:

- Diagnosis of a current unipolar depressive episode according to DSM-IV criteria
- Prescription of antidepressant treatment - SSRI or α2-antagonist - by the psychiatrist or referring physician for the current depressive episode
- Age 18 or older
- Affiliation with a welfare scheme and native French speakers
- Complete information on the study received and written informed consent signed

The exclusion criteria are as follows:

- Diagnosis of a progressive psychiatric disorder (other than MDD and anxiety disorder) according to DSM-IV criteria
- Seasonal characteristics of the depression
- Current antidepressant treatment or in the 6 months preceding the start of the study
- Recommended antidepressant treatment other than SSRI or α2-antagonist
- High suicide risk
- Retinal or ophthalmologic pathology affecting visual acuity as assessed by the Monoyer scale.
- History of head trauma, epilepsy or other neurological disorders
- Participation in another interventional study (including the exclusion period)
- Intellectual disability leading to difficulty participating or inability to understand the information provided in the study.
- Persons cited in Articles L. 1121–5 to L. 1121–8 of the French Public Health Code: pregnant women, parturient or breastfeeding mothers, persons deprived of their liberty by a judicial or administrative decision, persons under psychiatric care under duress, persons admitted to a health or social establishment for goals other than research, minors, adults subject to a legal protection, adults who are unable to express their consent and who are not subject to a legal protection measure.
- Criteria incompatible with the use of the ERG device: open wound in an area covered or enveloped by the device, implantable medical device (e.g. pacemaker), patient with a contagious disease

### 2.4 Procedure

**Figure 1:**
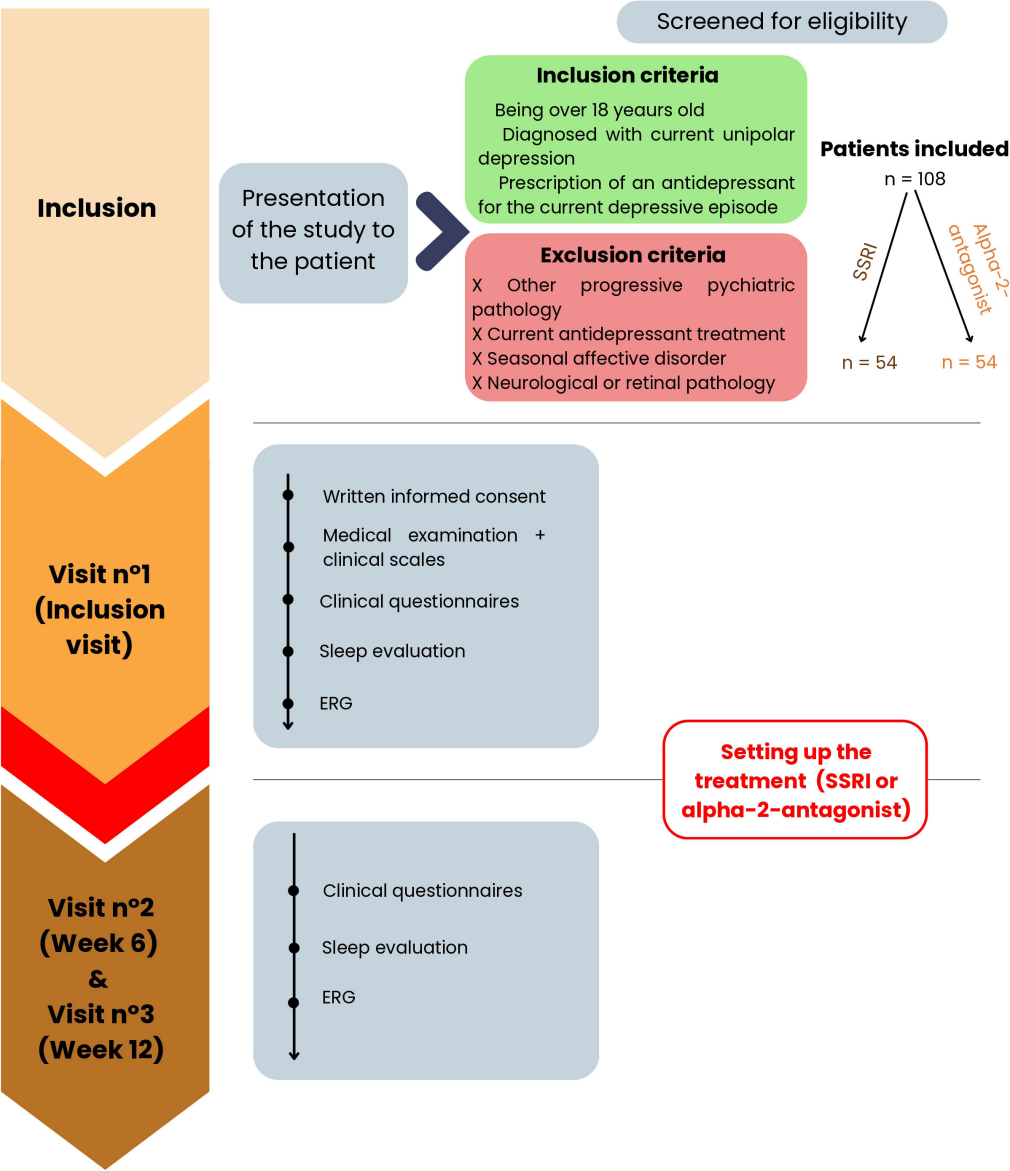
Flow diagram of the MESANTIDEP study. One hundred and eight patients will be included in the trial. They are divided into two groups according to the prescription of their first-line antidepressant: a SSRI or an α2-antagonist. The study consists of two phases. The first one is the inclusion visit when the study is presented to the patient. It allows tests to be carried out before taking the antidepressant. The next day, patients can start taking their prescribed antidepressant. The second phase of the study is the phase with antidepressants when the same tests are performed at 6 and 12 weeks after the start of the treatment. ERG, ElectroRetinoGraphy; SSRI, Selective Serotonin Reuptake Inhibitor; W6, Week 6; W12, Week 12.

A flow diagram of the study is provided in **Figure 1**. The following sections present the details of the interventions and evaluations.

The study will be split into two phases. The first phase is the pre-treatment phase, corresponding to the inclusion visit on Day 0 (D0). At the end of the inclusion visit, the patient can begin their SSRI or α2-antagonist treatment depending on their prescription and thus, their group. The second phase is the phase with antidepressants, during which patients take their antidepressant according to their clinician’s recommendations. This phase starts immediately after the first phase and lasts 12 weeks. The total duration of the study per patient adds up to 12 weeks.

#### 2.4.1 Inclusion visit

Once the patients’ eligibility has been verified, they can start participating in the study. During the inclusion visit at D0 and before any examination or act specific to the study, the investigator informs the participant about the study and obtains their free, informed and written consent. Then, a clinician collects socio-demographic and medical data. The clinical assessment is completed by a physical examination measuring height, bodyweight and visual acuity as well as a retrospective consumption diary (alcohol and tobacco). Finally, clinical scales and questionnaires evaluating “trait” and “state” markers of the disease are submitted to patients. Clinical scales - the AUDIT and Fagerström test - are used to check for the absence of other psychiatric disorders whereas clinical questionnaires - the MADRS and Hamilton Anxiety Scale - assess the intensity of depression. A sleep evaluation is also carried out thanks to questionnaires. The clinical assessment is based on an interview lasting approximately 1:30h.

In a second step, participants will perform an ERG recording in Nancy using the MonPackOne^Ⓡ^ device developed by metrovision and in Paris using the RETeval^Ⓡ^ developed by LKC Technologie. Both devices comply with International Society for Clinical Electrophysiology of Vision (ISCEV) standards. The duration of this exam is approximately 1 hour.

The patient inclusion visit will be carried out at the Nancy Psychotherapeutic Center or the Bichat Claude-Bernard Hospital, depending on the inclusion center. It is estimated to last 2:30h.

#### 2.4.2 Phase with antidepressants

Two visits are planned, 6 weeks (W6) and 12 weeks (W12) after the baseline evaluation. Indeed, the clinical efficacy of antidepressants is generally observed between 6 and 12 weeks (19).

During these visits, all the assessments above - except the socio-demographic and clinical data collection as well as the clinical scales - will be carried out for all the subjects. The Medication Adherence Rating Scale (MARS) to treatments is added for these two visits. The duration of each visit is estimated at 2 hours.

### 2.5 Devices characteristics

This study does not aim to assess the efficacy or safety of any particular product or device. However, it is based on electrophysiological ERG measurements. The devices used to collect the electrophysiological measurements required for our study are as follows:

#### 2.5.1 MonPackOne^Ⓡ^

At the Nancy Psychotherapeutic Center, ERGs are performed with the MonPackOne^Ⓡ^ system (Metrovision, Perenchies, France). This device is ISCEV compliant (20), MDA-approved and CE-marked. It allows the recording of pattern, full-field and multifocal ERGs using active corneal electrodes - Dawson, Trick and Litzkow (DTL) - and ground skin electrodes. With this device, the pupils are generally dilated thanks to a mydriatic. However, to reduce the discomfort and risks associated with pupillary dilation, pupils are not dilated with the MonPackOne^Ⓡ^ in the MESANTIDEP study. This is less restrictive for patients - in particular, because they can drive after the ERGs have been performed, unlike when pupillary dilation is used - reducing the proportion of loss to follow-up and increasing inclusions.

#### 2.5.2 RETeval^Ⓡ^

At the Bichat Claude-Bernard hospital in Paris, ERGs are performed using the RETeval^Ⓡ^ system (LKC Technologies, Gaithersburg, USA). This is a portable, ISCEV-compliant (20), MDA-approved and CE-marked device. It allows the recording of full-field ERGs with Sensor Strip^Ⓡ^ skin electrodes. This device is non-mitriatic.

### 2.6 Measures and outcomes

#### 2.6.1 Primary outcomes: ERG measurements and MADRS

All the measurements performed during this study are summarized in **Table 1**. As the primary outcome measures, we chose ERG measurements at D0 and the differences in the MADRS (21) scores between baseline and the 12th week of the study.

**Table 1:** Schedule of measurement and assessments for the MESANTIDEP study.

Assessments		Study period			
		24 to 48H before inclusion	Inclusion visit *(D0)*	Visit n°2 *(Week 6)*	visit n°3 *(Week 12)*
Project information		X			
Written informed consent			X		
Eligibility assessment			X		
Medical examination	*Anamnesis*		X		
	*Socio-demographic and medical data collection*		X		
	*Physical examination (Height, bodyweight, visual acuity)*		X		
	*Retrospective consumption diary (tobacco and alcohol)*		X	X	X
Clinical scales	*AUDIT, Fagerström test*		X		
Clinical questionnaires	*MADRS, MADRS-self, HAM-A*		X	X	X
	*GDS (for patients > 65 years old)*		X	X	X
	*MARS*			X	X
Sleep evaluation	*PSQI, ESS, ISI*		X	X	X
ERG	*ffERG (both centers)* *PERG, mfERG (Nancy)*		X	X	X

The names of the measures and assessments carried out in the MESANTIDEP study are detailed. The table also illustrates the visit when each assessment is conducted.

*AUDIT, Alcohol Use Disorders Identification Test; D0, Day 0; ESS, Epworth Sleepiness Scale; ERG, ElectroRetinoGraphy; ffERG full-field ERG; GDS, Geriatric Depression Scale; HAM-A, Hamilton Anxiety Rating Scale; ISI, Insomnia Severity Index; MADRS, Montgomery and Asberg Depression Scale; MARS, Medication Adherence Report Scale; mfERG, Multifocal ERG; PERG, Pattern ERG; PSQI, Pittsburgh Sleep Quality Index.*

**Figure 2:**
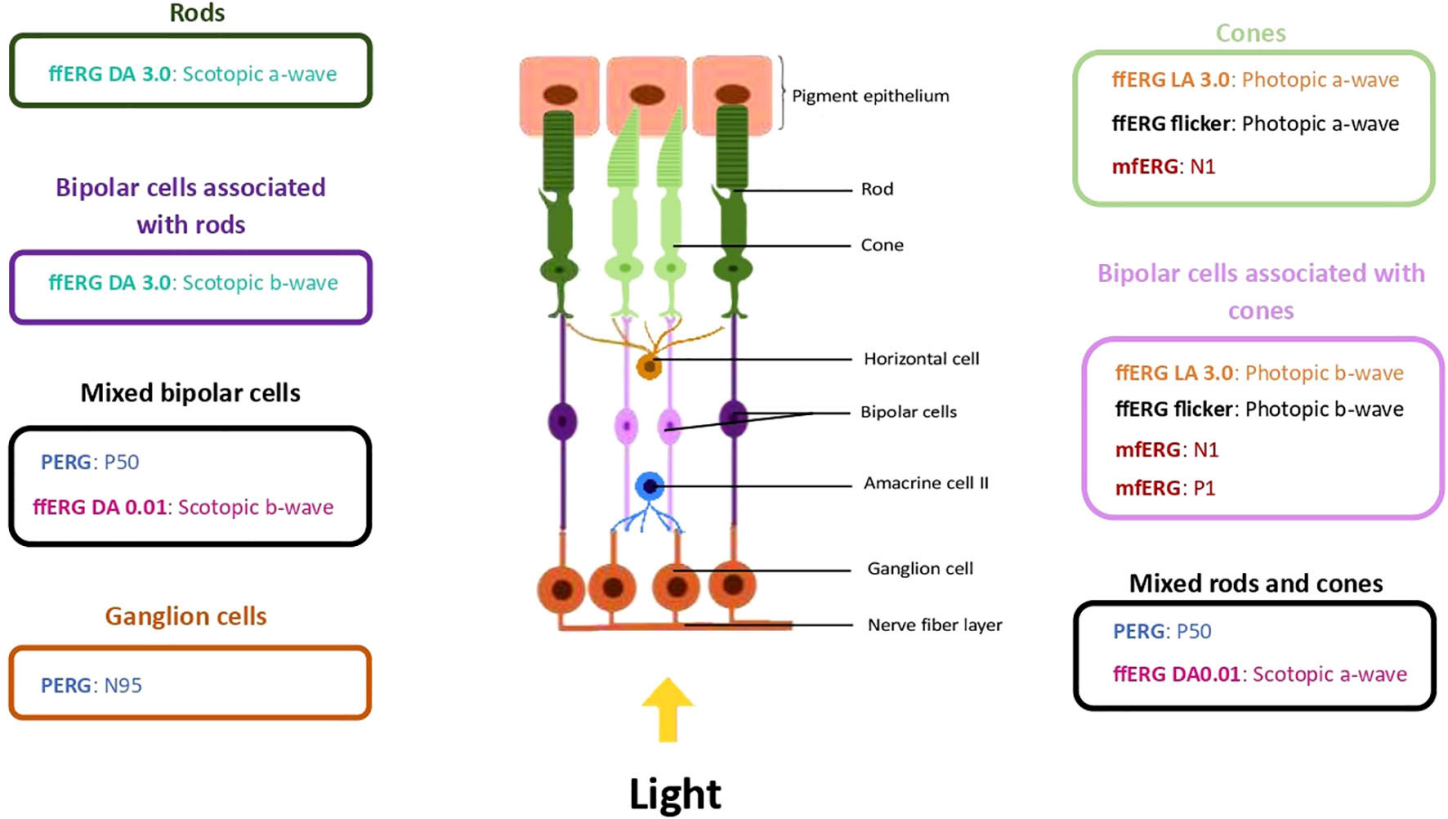
Characteristic ERG waves and their retinal associated cells. In the figure, waves analyzed in ffERG, PERG and mfERG are associated with the retinal cells they represent. The ffERG DA 3.0 waves are represented in green, the ffERG LA 3.0 waves in orange, the ffERG DA 0.01 waves in rose, the ffERG flicker waves in black, the PERG waves in blue and the mfERG waves in red. ERG, ElectroRetinoGraphy; ffERG, full-field ERG; mfERG, Multifocal ERG; PERG, Pattern ERG.

Retinal function will be assessed thanks to ERG measurements carried out at inclusion with MonPackOne^Ⓡ^ (Metrovision, Perenchies, France) in Nancy and with RETeval^Ⓡ^(LKC Technologies, Gaithersburg, USA) in Paris. In Nancy, electrophysiological measurements will be performed using full-field ERG (ffERG), pattern ERG (PERG) and multifocal ERG (mfERG). In Paris, electrophysiological measurements will be performed using ffERG. In both centers, sequences used for ffERG are ffERG DA 0.01, DA 3.0 and LA 3.0 and flicker. The parameters recorded will be the amplitude - in microvolts - and the implicit time - in milliseconds - of waves of interest, according to test sequences. The ffERG allows recording of the peripheral retina, with a minimal contribution from the macula (20). To that end, different flashes of light are delivered under a dark background - in scotopic conditions - or under a light background - in photopic conditions (22). The waves obtained are the a-wave - representative of rods in scotopic conditions and cones in photopic conditions - and the b-wave - reflecting inner retinal glia and bipolar cells - (23). The PERG allows recording of the macula thanks to a black and white checkerboard pattern alternating (24). The characteristic waves analyzed are P50 (especially photoreceptors and bipolar cells) and N95 (ganglion cells) (24). The mfERG allows topographical representation and localization of retinal activity under photopic conditions (25). To that end, stimuli are a set of graduated hexagons alternating between light and dark states (25). Waves obtained are N1 (cone bipolar cell function with cone participation) and P1 (cone bipolar cell function) (25). The correspondence between ERG waves and the associated retinal cells is summarized in **Figure 2**.

MDD symptoms severity will be assessed thanks to MADRS scores at inclusion and after 12 weeks. The MADRS items are rated on a 4-point Likert scale from 0 (no distress) to 3 (maximum distress), with 1/2 points in between. The patient is asked to respond to the questionnaire by rating the number that best corresponds to their psychological state over the last three days. The total score ranges from 0 to 27. A score of 12 or less corresponds to the absence of a depressive episode, a score ranging from 13 to 19 to a mild depressive episode, and a score over 20 to a moderate or major episode of depression (21). The difference in MADRS scores between D0 and W12 will then be calculated for each patient to determine whether or not they are responding to their antidepressant treatment. The subject will be declared responder - if a decrease greater than or equal to 8 points is observed in the MADRS score - or non-responder - if the difference in MADRS score is less than 8 points or if an increase is observed (26).

#### 2.6.2 Secondary outcomes

##### 2.6.2.1 ERG measurements at W6 and W12

ERG measurements are taken not only at the inclusion visit but also at visit n°2 (W6) and visit n°3 (W12). Thus, the ERG parameters - amplitude and implicit time of characteristic waves - obtained in ffERG (for both centers), PERG and mfERG (for inclusions in Nancy) at W6 and W12 are used as secondary outcomes. The method used remains the same as for the inclusion visit (See §2.6.1).

##### 2.6.2.2 Anxiety and other depressive symptoms measurements

Depressive symptoms severity, as a secondary outcome, will be assessed thanks to MADRS scores at D0, W6 and W12.

To complete MADRS scores, depressive symptoms severity will also be assessed at inclusion and after 6 and 12 weeks thanks to MADRS-self scores for all patients and thanks to the Geriatric Depression Scale (GDS) for patients aged over 65. The principle of the MADRS-self scale is the same as MADRS, but patients complete the questionnaire themselves (21). The GDS is a 15-question hetero-questionnaire. For each of these questions posed by the clinician, the patient must answer “yes” or “no”. One of these options adds one point to the total score, while the other adds no points per question. According to the question, the point can be associated with the “yes” or the “no” answer. The total score ranges from 0 to 15. A score of 5 or less corresponds to the absence of a depressive episode, from 5 to 10 to a mild or moderate depressive episode, and over 11 to a major episode of depression (27).

In parallel with depressive symptoms, anxiety symptoms will be assessed based on the Hamilton Anxiety Rating Scale (HAM-A) at inclusion and after 6 and 12 weeks. This 14-item scale yields a total score ranging from 0 to 30. A score below 17 indicates average anxiety, between 18 and 24 an average to moderate anxiety, and over 25 a moderate to severe anxiety (28).

##### 2.6.2.3 Sleep settings

Another secondary outcome measure is sleep quality assessed at baseline and after 6 and 12 weeks based on three questionnaires. The first one is the Pittsburgh Sleep Quality Index (PSQI). It is a 7-item self-assessment scale investigating subjective sleep quality during the past month. A score over 5 indicates a disturbed sleep (29). The other two scores used are the Epworth sleepiness scale (ESS) and the Insomnia Severity Index (ISI). They evaluate, respectively, the daytime sleepiness and severity of insomnia. The ESS is an 8-item self-assessment scale with a total score rated out of 24. A score of at least 15 indicates excessive daytime sleepiness (30). The ISI is a 7-item self-assessment scale. It gives a total score ranging from 0 to 28. A score of 7 or less indicates the absence of insomnia. A score from 8 to 14 corresponds to a mild subclinical insomnia, from 15 to 21 to a moderate clinical insomnia, and over 22 to a severe clinical insomnia (31).

##### 2.6.2.4 Assessment of treatment compliance

Finally, patients’ compliance of their antidepressants will be assessed by the MARS. This 5-item scale relates to antidepressant treatment habits during the past month. Each item is marked from 1 to 5, and the total score ranges from 5 to 25. A score below 21 indicates that the patient is non-compliant with their treatment, whereas a score of 21 or more indicates that the patient is compliant (32).

##### 2.6.2.5 Baseline assessment

At the inclusion visit, an interview is performed to collect socio-demographic data (age, gender and level of academic achievement) and consumption habits (tobacco consumption in cigarettes per day and alcohol consumption per week, a retrospective consumption diary for tobacco, alcohol and concomitant treatments). The retrospective consumption diary is completed at every visit. For patients who smoke, the level of tobacco addiction is assessed using the Fagerström test (33). Alcohol abuse is evaluated by means of the Alcohol Use Disorder Identification Test (AUDIT) (34).

Then, a clinical and physical examination is conducted to obtain a general and surgical medical history, the height, the bodyweight and the visual acuity using the Monoyer chart. Patients should have a normal or best-corrected visual acuity to perform ERGs at the end of the inclusion visit. Other primary and secondary outcomes, except the MARS, are carried out as described above. All scales are completed using paper and pencil.

Finally, the clinician collects psychiatric assessment data.

### 2.7 Data analysis

#### 2.7.1 Data management

Data collection and management will comply strictly with current French legislation. Study data will be collected by the investigator or a designated person, and will be recorded directly in a research-specific observation notebook assigned to each participant. All the data obtained will be pseudonymized. Then, the data will be entered into a database hosted on the Nancy Psychotherapeutic Center secure internal network, and will be accessible only to investigators. Furthermore, all data presented in publications will be entirely anonymous, preserving the confidentiality of the patients included. At the end of the study, all the documents will be archived and kept for 15 years. After analysis of the data collected, a communication of the results related to this research may be offered to all participants.

#### 2.7.2 Sample size

To date, there are no combined studies in the literature on ERG parameters in patients with MDD before and during treatment providing statistics or parameters of measurement variability between D0 and W6. The percentage of non-responders can be estimated for the two therapeutic classes (SSRIs and α2-antagonists) at 38% ± 8% (i.e. between 30% and 46%). In order to obtain 38% of non-responders for the study with a confidence interval from 0.278 to 0.482, the number of subjects required is 96 at a risk of 5% (Simple Asymptotic Method with Continuity Correction). With the 12% loss to follow-up or whose data could not be used, the number of subjects to be recruited is 108, i.e. 54 subjects per group, which corresponds to our recruitment capacity.

#### 2.7.3 Statistical analysis

The results obtained in the MESANTIDEP study will be analyzed using two approaches.

In the first approach, ERG parameters will be considered independently, i.e. the amplitude and latency of each wave will be compared between two populations. This will allow to extract preliminary ERG markers. With this approach, the parameters collected are described by the usual indicators according to their nature: i) percentage and frequency for qualitative variables and ii) mean ± standard deviation or median and interquartile range for quantitative variables. For qualitative variables, the chi-squared test or the Fisher’s exact test will be performed according to the number of patients for each group and modality. For quantitative variables, the normality of distributions and the equality of variances will be assessed by a Shapiro-Wilk test and a Levene test respectively. Then, comparisons of outcomes between the two groups are performed using the Student’s t-test on paired series if a parametric test is necessary or using the Mann-Whitney U test if data are not parametric. The Pearson’s coefficient or the Spearman’s rho will allow analysis of the correlations. Intermediate data analysis is planned 9 months (mid-term study) after the start of inclusions. The statistical significance level is set at 0.1% for the interim analysis and 4.9% for the final analysis. Missing values will not be imputed. If the number of missing values becomes relevant (main outcome, number of missing data, imbalance, etc…), a possible method for imputing medical data may be considered and must be justified in the statistical analysis plan.

Then, at the end of the study, once all the data has been collected, results will be analyzed in collaboration with mathematicians specialized in signal processing. This will enable to extract more robust ERG markers via the development and use of mathematical signal processing techniques and machine learning algorithms. Indeed, with this approach, different variables, particularly those that can influence results, will be taken into account in the analysis of results, enabling groups to be homogenized.

#### 2.7.4 Safety monitoring

As the MESANTIDEP study includes only safe tests and evaluations, no specific adverse events or effects are expected and thus no particular risk is expected. If a Serious Adverse Event (SAE) occurs, it will automatically be considered unexpected. The electrophysiological parameters of the ERG are measured by routine, non-invasive and well-tolerated examinations. When measuring ERG with the MonPackOne^Ⓡ^ device, the use of DTL electrodes (conductive wires placed in the conjunctival cul-de-sac) does not require local anesthetic. There is a minimal risk of conjunctival irritation, which is brief, transient and resolves spontaneously. The investigators have a good experience in the use of the different devices. Subjects with epilepsy will be excluded from the study because of the repeated light stimulation with ERGs. We do not expect an effect of the study on the course of MDD for patients. The investigators will systematically question all participants during various visits to look for possible adverse events. The presence or absence of adverse events will be recorded in the study’s case report form at each study visit. If an adverse event has occurred from the date of inclusion and throughout the duration of the study, it will be declared without delay according to the French usual reporting procedure to the concerned health vigilance institution. Anyone with an adverse event will receive treatment appropriate for their condition and will be followed until the event is resolved or until the end of the research. If necessary, the experienced device or test will be stopped for that person.

#### 2.7.5 Duration of the study and stopping rules

The MESANTIDEP study consists of three visits over a 12-week period for each patient. The total planned inclusion period is 15 months.

Drop-outs from the study are permanent. After confirmation by the investigator and the promoter, they occur: i) when the patient wishes to discontinue their participation in the study (with or without withdrawal of consent), ii) with a non-compliance with the framework of the care service, as defined by its internal regulations, or iii) with the onset of a SAE justifying study termination. Any patient may stop participating in the research at any time and for any reason without incurring any liability or prejudice as a result. The investigator may temporarily or permanently discontinue an individual’s participation in the research for any reason that affects the patient’s safety or would be in the patient’s best interest. A drop-out of the study, for any reason, will not influence the quality of care that will be provided to the patient and will not affect their medical care. Given the nature of the study, no special follow-up is required at the end of participation in this study. In cases of study withdrawal (examination(s) not performed or withdrawal of consent), the patient is replaced by another one of the same group (patient with a SSRI or with an α2-antagonist).

## 3 Discussion

### 3.1 Hypothesis

**Figure 3:**
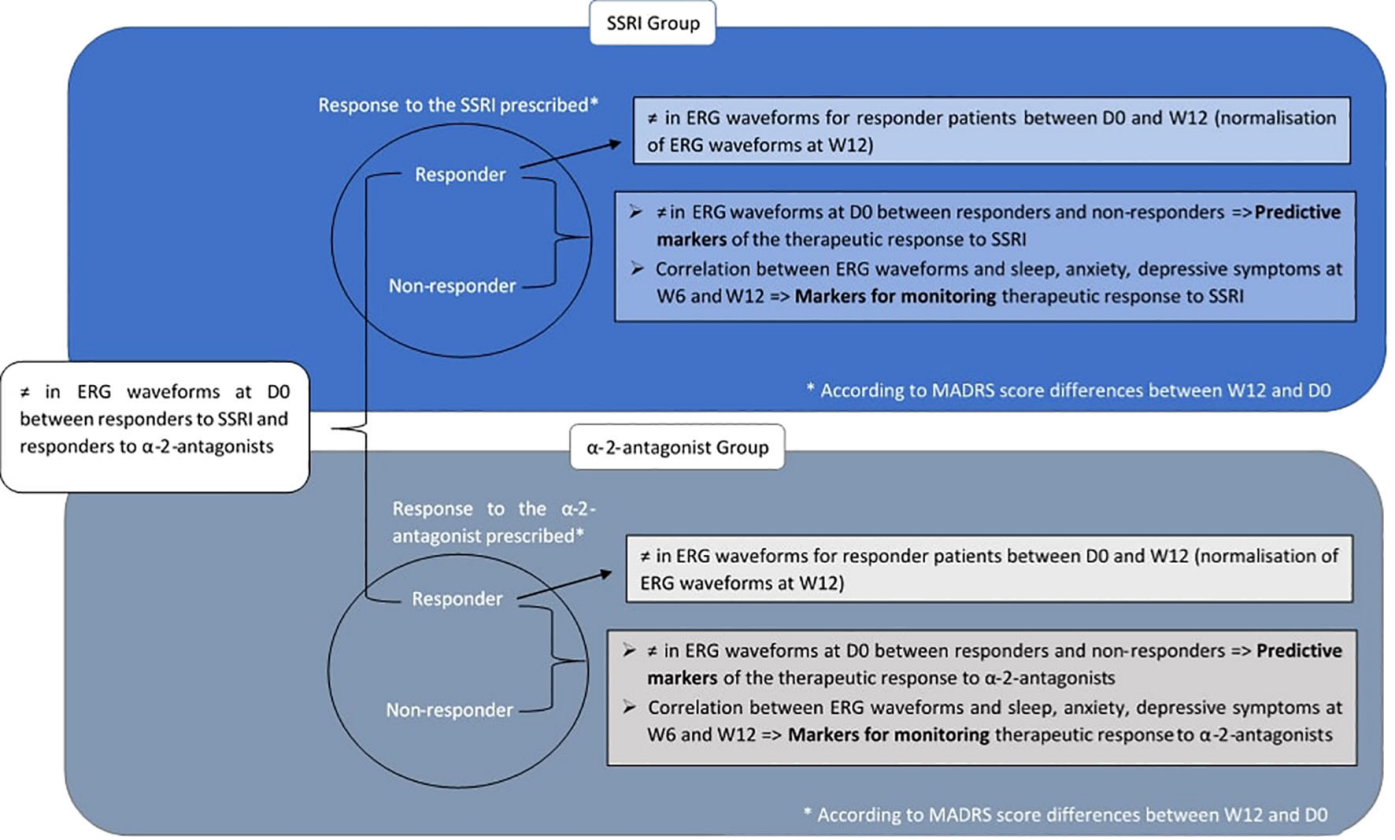
Comparisons of ERG waveforms planned for MESANTIDEP data analysis and associated expected results. Comparisons of ERG waveforms between and within patient groups planned for MESANTIDEP data analysis are summarized. Their associated expected results and their potential future use in psychiatry are annotated next to each comparison. ERG waveform results for the SSRI-treated MDD patients are boxed in dark blue, while those for the α2-antagonist treated MDD patients are boxed in gray. Within each patient group, responders and non-responders to their prescribed antidepressant are distinguished. This distinction will be based on the difference in MADRS score between W12 and the inclusion visit: the patient will be considered as responder - if a decrease greater than or equal to 8 points is observed in the MADRS score - or non-responder - if the difference in MADRS score is less than 8 points or if an increase is observed. D0 = Inclusion visit; MADRS Montgomery and Asberg Depression Scale; SSRI, Selective Serotonin Reuptake Inhibitor; W12, Week 12; ≠, differences.

The results of the MESANTIDEP study will improve our understanding of the effects of antidepressants on retinal function according to their pharmacological class. Comparisons of ERG waveforms between and within patient groups as well as their associated expected results when analyzing MESANTIDEP data are summarized in **Figure 3**.

First, at the end of the study, MADRS scores will be compared between the inclusion visit and W12 for each patient. This will enable the differentiation of responder patients from non-responder patients within the same group. Patients will be declared responders if a decrease greater than or equal to 8 points is observed in the MADRS score between D0 and W12 (26). ERG measurements - amplitudes and implicit times of characteristic ERG waves - at D0 of responders in the SSRI group will be compared with those of non-responders in the same group. The same comparison will be performed for the α2-antagonist group. We hypothesize that significant differences could be observed for ERG measurements between responders and non-responders of the same antidepressant group. According to the study by Moulard et al. (35), SSRI responders should show an increase in a- and b-wave amplitudes in photopic ffERG as well as a decrease in the implicit time of the P50 wave in PERG. A significant decrease of the scotopic b-wave could also be observed in responders to duloxetine compared to non-responders (36). To date, there are no studies on the specific effects of α2-antagonists on ERG. It is therefore difficult to know what differences could be expected precisely in ERG between responders and non-responders to α2-antagonists. These potential effects of SSRIs and α2-antagonists on ERG waveforms would be explained by their action on monoamine pathways, notably on dopamine and serotonin as detailed in the review by De Deus et al. (17). The decreased amplitude of the photopic b-wave in ffERG expected in SSRI responders would be caused in particular by decreased dopaminergic receptors and retinal dopamine.

Similarly, the HAM-A and sleep scales - ESS, ISI and PSQI - will be compared at D0 between responders and non-responders to the same antidepressant class. We hypothesize that significant differences will also be present with these scales, HAM-A and sleep scales scores being higher in non-responders at D0. Indeed, anxious and sleep problems have been associated with non-response to antidepressants (37, 38).

Another hypothesize is that significant differences would exist between ERG parameters at D0 of SSRI responders and α2-antagonist responders. Indeed, SSRIs and α2-antagonists do not have the same mechanisms of action. SSRIs inhibit the serotonin transporter (SERT), which in turn inhibits the presynaptic reuptake of serotonin. As a result, the level of serotonin in the synaptic cleft rises, increasing post-synaptic stimulation of serotonin receptors (39). α2-antagonists mainly inhibit presynaptic alpha-2 adrenergic receptors, resulting in an increase in noradrenaline release, which would indirectly increase serotonin release (40, 41). Thus, the two classes of antidepressants studied in MESANTIDEP - SSRIs and α2-antagonists - do not have the same molecular targets. A responder to SSRIs will therefore not have the same molecular cause of their depressive episode as a responder to α2-antagonists: a patient responding to SSRIs will probably present a dysfunction of their SERT, whereas for a patient responding to α2-antagonists it will be rather at the level of α2-adrenergic receptors. These differences in dysfunctions would lead to differences in ERG profiles between SSRI responders and α2-antagonist responders. This hypothesis can be supported by the fact that the results concerning certain amplitudes and implicit times of the electrophysiological waves of interest may differ according to the therapeutic class administered in humans (17).

Correlations between the ERG and MADRS scores will then be calculated at D0, W6 and W12 for the SSRI and α2-antagonist groups. This will allow checking whether the ERG results normalize as the MADRS scores decrease, as shown previously (42). The same analysis will be carried out between both HAM-A scores and sleep questionnaires - the ISI, ESS and PSQI - with ERG measurements. The hypothesis is that there is a correlation between these clinical scales and ERG parameters. Indeed, these scales assess clinical symptoms generally associated with depressive episodes. Thus, such as MADRS scores, the ERG could be normalized as those symptoms improve.

### 3.2 Future directions

In the near future, if the MESANTIDEP results are promising, a similar study could be carried out with other antidepressant classes such as Serotonin-Norepinephrine Reuptake Inhibitors and Tricyclic Antidepressants.

If the hypotheses proposed above (§3.1) prove to be validated in the MESANTIDEP study and in other similar studies, ERG data could represent valuable tools for psychiatric clinicians. First, ERG differences could be found between responders and non-responders to the same therapeutic class before the start of the antidepressant treatment. In this case, these ERG parameters, shown as different, could constitute predictive markers of the therapeutic response to the class of antidepressant of interest. ERG parameters could also constitute markers for monitoring therapeutic response if they are shown to be correlated with clinical scales - MADRS, HAM-A, ISI, ESS and PSQI. If certain ERG parameters correlate with HAM-A or sleep scales (ISI, ESS and PSQI), then these parameters could also reflect anxiety or sleep disorders respectively in MDD patients. In this case, it would be possible to propose an adjunctive non-pharmacological treatment more adapted to the patient, such as psychological follow-up for anxiety patients, or light therapy or lifestyle intervention for patients with sleep disorders (43).

Since ERG measurements are reliable, objective and reproducible, they could pave the way toward precision medicine, where each individual could benefit from an adapted and personalized treatment (18). To achieve this goal, multimodal approaches are preferred over isolated markers (44). For this reason, the MESANTIDEP study also evaluates clinical anxiety and sleep scales as potential markers for predicting therapeutic responses to antidepressants. Other studies suggest the presence of markers of responses to antidepressants in blood (45) or through neuroimaging (46). Together with ERG measurements, these markers could be used to define profiles of patients who may or may not respond to a particular class of antidepressant. This will allow better tailoring of the first-line antidepressant prescription. Then, ERG markers could be used to monitor the response to this antidepressant, to know if and when it needs to be changed. This multimodal approach would enable therapeutic intervention to be adapted more rapidly to each individual, thereby limiting the functional impact and reducing the morbidity and mortality associated with the disease (44).

### 3.3 Limitations and strengths of the study

#### 3.3.1 Limits

However, the MESANTIDEP study has certain limitations.

First, because of its multicenter approach, a center effect could be observed between ERG data. Indeed, the devices used for ERG measurements are not the same between centers - RETeval^Ⓡ^ for the Paris center and MonPackOne^Ⓡ^ for the Nancy center. Despite this difference, the procedure remains the same, as the pupils are not dilated and the visual stimuli are the same, both devices being based on ISCEV standards. Nonetheless, the electrodes used are not the same - Sensor Strip^Ⓡ^ skin electrodes for Paris and DTL electrodes for Nancy - nor are they placed in the same places. This could influence the ERG measurements and thus create differences between center results (47, 48). Moreover, ERGs are carried out by different sensors, which is a well-known perturbating factor in difference detection (49). For these reasons, during the statistical analyses, the absence of a center effect will be checked. The multicenter approach has advantages, however, since it provides robustness, as it enables the recruitment of a larger number of patients and from different geographical areas.

Another limitation of the MESANTIDEP study concerns the treatments taken by the included patients. Different molecules are available within the same therapeutic class, and therefore within the same group of patients. Even though the primary mechanism of action of antidepressants within the same class is similar, each SSRI (50) and α2-antagonist (51) antidepressant has its own unique pharmacological properties, which could lead to a molecular effect on the ERG results. However, to our knowledge, there are currently no studies showing differences in ERG waveforms between different antidepressants of the same class. To ensure that all antidepressants in the same class have similar effects on ERG measurements, ERG waveforms comparisons will be done between each antidepressant within the same class (intra-group comparisons) if the number of patients allows it.

In addition, patients may receive other treatments than antidepressants, such as atypical antipsychotics, anxiolytics or lithium carbonates. These drugs have been shown to cause differences in ERG (52–57). Apart from treatment, other substances taken by patients may influence ERG results. This is the case with illicit drugs (58–62), which is why patients currently taking any illicit drug, even occasionally, will not be included in the study. However, patients using tobacco and/or alcohol, if there are no associated with behavioral problems, may be included in the study. Otherwise, recruitment of MDD patients would be much more difficult. Moreover, the effect of tobacco and alcohol in ERG has been well documented in the scientific literature (63–67). These litterature data, combined with those collected during the study - notably by the retrospective consumption diaries, AUDIT and Fagerström test - will allow to consider these consumption data as variables to take account when analyzing ERG data. Thus, the different groups of patients will be balanced to avoid differences between each group with regard to treatments.

Finally, the choice of the antidepressant prescribed depends mainly on the patient’s clinical profile - in particular, medical and psychiatric co-morbidities, severity of the depression and symptom profiles - but also on many other factors such as treatment response in past episodes, patient and clinician preference, treatment availability, likelihood of adhering to treatment and possible side effects (43, 68). Therefore, there is a risk of significant differences between the profiles of SSRI treated and α2-antagonist treated patients, making inter-group comparisons complicated and not very robust. However, when analyzing the results, most comparisons will be made between patients in the same group. The only inter-group comparison to be made will be between the ERG waveforms of SSRI responders and those of α2-antagonist responders at inclusion (§3.1). The aim of this comparison is precisely to show that there are cerebral differences between patients effectively treated with a SSRI and those effectively treated with an α2-antagonist. In addition, to avoid other differences between the two groups influencing the results, a mathematical method considering all variables will be used, as detailed in the statistical analysis section (§2.7.3).

#### 3.3.2 Strengths

In contrast, the MESANTIDEP study also has strengths, particularly in terms of feasibility. As the two study centers are located in hospitals with a large network of practitioners, recruitment should be easier. This will enable the recruitment of many patients, which will increase the robustness of the study.

In addition, we are a multidisciplinary team specializing in ERGs and have already carried out various studies on this tool and psychiatric disorders (CAUSAMAP: NCT02864680, BiMAR: NCT05161546; LUMIDEP: NCT03685942, REVIPSY: NCT05167396, ERICA: NCT03818971). Therefore, we will be able to interpret the ERG results more easily and resolve any problems that may arise during the MESANTIDEP study.

Finally, one of the specificities and strengths of the MESANTIDEP study lies in the innovative nature of its subject. Indeed, only a few studies have assessed the link between antidepressants and retinal electrophysiology. Among them, only four focused on the effects of antidepressant classes (35, 36, 69, 70) and none of them focused on α2-antagonists. However, the MESANTIDEP study is longitudinal, unlike these studies, which makes it possible to observe the effects of antidepressant classes over time. Patients are also their own controls between D0, when they are not taking their treatment, and W6 and W12, when their treatment has started. This removes the bias of individual factors - such as age, sex and treatments - which can influence ERG results (§3.3.1 71–74).
